# Semiautomatic Assessment of Facet Tropism From Lumbar Spine MRI Using Deep Learning: A Northern Finland Birth Cohort Study

**DOI:** 10.1097/BRS.0000000000004909

**Published:** 2023-12-18

**Authors:** Narasimharao Kowlagi, Antti Kemppainen, Egor Panfilov, Terence McSweeney, Simo Saarakkala, Mika Nevalainen, Jaakko Niinimäki, Jaro Karppinen, Aleksei Tiulpin

**Affiliations:** a Research Unit of Health Sciences and Technology, University of Oulu, Oulu, Finland; b Department of Diagnostic Radiology, University Oulu Hospital, Oulu, Finland; c Rehabilitation Services of South Karelia Social and Health Care District, Lappeenranta, Finland; d Neurocentral Oulu, Oulu University Hospital, Oulu, Finland

**Keywords:** facet joints, facet tropism, deep learning, zygapophyseal joints, facet asymmetry

## Abstract

**Study Design.:**

This is a retrospective, cross-sectional, population-based study that automatically measured the facet joint (FJ) angles from T2-weighted axial magnetic resonance imagings (MRIs) of the lumbar spine using deep learning (DL).

**Objective.:**

This work aimed to introduce a semiautomatic framework that measures the FJ angles using DL and study facet tropism (FT) in a large Finnish population-based cohort.

**Summary of Data.:**

T2-weighted axial MRIs of the lumbar spine (L3/4 through L5/S1) for (n=1288) in the NFBC1966 Finnish population-based cohort were used for this study.

**Materials and Methods.:**

A DL model was developed and trained on 430 participants’ MRI images. The authors computed FJ angles from the model’s prediction for each level, that is, L3/4 through L5/S1, for the male and female subgroups. Inter-rater and intrarater reliability was analyzed for 60 participants using annotations made by two radiologists and a musculoskeletal researcher. With the developed method, we examined FT in the entire NFBC1966 cohort, adopting the literature definitions of FT thresholds at 7° and 10°. The rater agreement was evaluated both for the annotations and the FJ angles computed based on the annotations. FJ asymmetry ( $\theta_{L}$ - $\theta_{R})$ was used to evaluate the agreement and correlation between the raters. Bland-Altman analysis was used to assess the agreement and systemic bias in the FJ asymmetry. The authors used the Dice score as the metric to compare the annotations between the raters. The authors evaluated the model predictions on the independent test set and compared them against the ground truth annotations.

**Results.:**

This model scored Dice (92.7±0.1) and intersection over union (87.1±0.2) aggregated across all the regions of interest, that is, vertebral body (VB), FJs, and posterior arch (PA). The mean FJ angles measured for the male and female subgroups were in agreement with the literature findings. Intrarater reliability was high, with a Dice score of VB (97.3), FJ (82.5), and PA (90.3). The inter-rater reliability was better between the radiologists with a Dice score of VB (96.4), FJ (75.5), and PA (85.8) than between the radiologists and the musculoskeletal researcher. The prevalence of FT was higher in the male subgroup, with L4/5 found to be the most affected region.

**Conclusion.:**

The authors developed a DL-based framework that enabled us to study FT in a large cohort. Using the proposed method, the authors present the prevalence of FT in a Finnish population-based cohort.

Low back pain (LBP) is one of the leading causes of years lived with disability.^1^ In 2017, it was estimated that about 576 million people were affected at any given time.^1^ LBP is characterized by complex etiology and is multifactorial.^2^ Common causes of LBP include disc degeneration (DD) and facet joint (FJ) osteoarthritis.^3^ Prior research suggests facet tropism (FT) as one of the suggested risk factors for DD.^4^ It was found that FT can alter the biomechanics of the intervertebral disc.^5^ An increased FT can cause increased intradiscal stress and can be a potential risk factor for lumbar disc herniation.^5^ Also, FT is known to increase the likelihood of degenerative lumbar spinal stenosis by ∼2.9 times.^6^

FT is defined as the difference between the left and right FJ angles.^7^ There is no universal threshold for FT^4,8–20^ (Table 1). This can be attributed to several factors, such as the study, that is, the threshold at which an association is seen with other lumbar spine disorders (LSD), sample sizes, and others. Prior research on FT was done using manual measurement of FJ angles. Therefore, the studies can be constrained to a few hundred subjects, as it is time-consuming and requires relevant expertise to reliably measure the angles at different spine levels for large populations. Using automated methods such as those based on deep learning (DL) can facilitate large-scale studies and help identify reliable insights from data with low human error, cost, and time.^21^

**TABLE 1:** Definition of Facet Tropism in the Prior Art

Year	References	Number of participants	Modality	Tropism definition
1993	Vanharanta *et al* ^8^	108	CT	Normal: <mean Moderate: mean+1×STD Severe: mean+2×STD
1999	Love *et al* ^13^	10	CT	—
2009	Kalichman *et al* ^14^	188	CT	>7°
2012	Chadha *et al* ^15^	60	MRI	>10°
2014	Bao *et al* ^16^	23	CT	>10°
2015	Schleich *et al* ^4^	25	MRI	>10°
2015	Samartzis *et al* ^17^	349	MRI	>7°
2016	Wang *et al* ^17^	65	MRI	>10°
2016	Samartzis *et al* ^19^	349	MRI	>8°
2016	Gao *et al* ^20^	242	MRI	>10°
2017	Zhou *et al* ^9^	129	CT	>5°
2017	Mohanty *et al* ^10^	124	CT	Mild: 5°–7° Moderate: 7°–15° Severe: >15°
2018	Ko *et al* ^11^	462	CT	>7°
2021	Ma *et al* ^12^	198	CT	—

The summary covers different imaging modalities and highlights the lack of a universal threshold for facet tropism.

CT indicates computed tomography; MRI, magnetic resonance imaging.

DL has been successfully used in spine image analysis for automatic segmentation and classification.^22–25^ It is shown that DL improves data processing efficiency with consistent results for lumbar spine magnetic resonance imaging (LSMRI).^21^ This work aims to introduce a DL-based method that can reliably scale and eventually replace the time-consuming manual measurements of FJ angles using routine T2-weighted axial LSMRI. We hypothesize that FT can be an important clinical biomarker that may further explain LSD such as DD. Therefore, we need a reliable method that can be efficiently applied to large and diverse cohorts. We consider our current work as a first step that can reliably automate the measurement of FT at scale and demonstrate very good agreement (*r* ^2^=0.84) between the radiologists for FJ asymmetry. The clinical utility of this framework can be further expanded by studying FT as a biomarker in relation to LSD using large cohorts.

## MATERIALS AND METHODS

### The Northern Finland Birth Cohort 1966

The Northern Finland Birth Cohort 1966 (NFBC1966) data set contains data from 12,231 participants born in 1966 and followed up at different time points.^26^ 1540 participants (age=46) underwent a LSMRI at Oulu University Hospital. Imaging was performed from 2011 through 2015 using a 1.5 Tesla GE Signa HDxt (General Electric, Milwaukee, WI, USA) scanner. The T2-weighted fast-recovery fast spin-echo acquisition was made for both Sagittal (TR/effTE 3500/112 ms, pixel spacing 0.5, slice thickness 3 mm, Field of view 280×280 mm, 512×512 pixel resolution) and Axial planes (TR/effTE 3600/118 ms, pixel spacing 0.3, slice thickness 4 mm, Field of view 180×180 mm, 512×512 pixel resolution). Axial imaging was only available from L3/4 through L5/S1. The participants provided informed consent, and the imaging adhered to the Declaration of Helsinki, approved by the Northern Ostrobothnia Hospital District Ethics Committee. The participant’s personal information was removed from the DICOM metadata. Participants also underwent clinical examinations and answered questionnaires related to LBP.^26^

### Data Selection and Annotations

**Figure 1:**
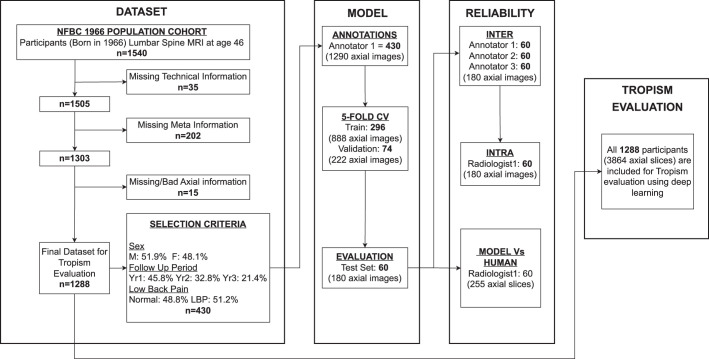
A schematic overview of the study. The study is divided into four steps. The data set block outlines the participants’ inclusion, exclusion, and selection criteria. The model block shows the participant subsets used for annotations, training, and evaluation methods. The reliability blocks show the data used for analyzing inter-rater and intrarater reliability and the agreement between the model predictions and the raters. Lastly, the tropism evaluation block outlines the participants used in the evaluation of asymmetry in NFBC1966.

In this study, a total of (n=1288) participants were chosen. The data selection and exclusion criteria for obtaining the participants for the FT evaluation are outlined in Figure 1. From the available participants, 430 individuals who underwent MRI between 2011 and 2015 were chosen while maintaining equal proportions for sex and LBP status. Participants with LBP were identified through self-reported questionnaires. Participants chose between “none,” “1 to 7 days,” “8 to 30 days,” “over 30 days,” and “daily” for LBP prevalence. The category “none” was considered for participants with no LBP, and the rest with LBP.

To evaluate FT, we used T2-weighted axial sequences. We identified three regions of interest (ROI) necessary to compute the angles: the vertebral body (VB), FJ, and the posterior arch (PA). As an initial step, WEASIS DICOM viewer^27^ was used to preselect the axial sequences corresponding to the given spine level. Although several slices exist for the given level, the annotator selected the slice with the best representation of the chosen ROIs for each level from the MRI volume. This was a deliberate choice because the available axial imaging was acquired for routine examination and was not optimized for FJ; that is, the anatomical structures required to compute FJ angles were not visible in all the slices. Using this process, the metadata was built for all the participants (n=1288), such that each participant had three axial slices.

Three annotators with diverse experience performed the required annotations. Annotators: A1 (AK—fourth-year resident radiologist) and A2 (MN—board-certified fellowship-trained musculoskeletal radiologist with 10 years of experience) are working at Oulu University Hospital. In contrast, annotator A3 (TM) is a MSK researcher with 10 years of experience as an osteopath. The initial set of 430 participants were annotated for 1290 axial slices by A1. The desired ROIs were annotated (pixel painting) to create a mask using ITK-SNAP (Version: 4.0.1, University of Pennsylvania, Philadelphia, Pennsylvania, USA) annotation software. A subset of 60 participants was chosen randomly as an independent test set. A2 and A3 performed a separate set of annotations for assessing inter-rater reliability, and A1 re-annotated the same subset after five months for intrarater reliability.

### Facet Tropism

**Figure 2:**
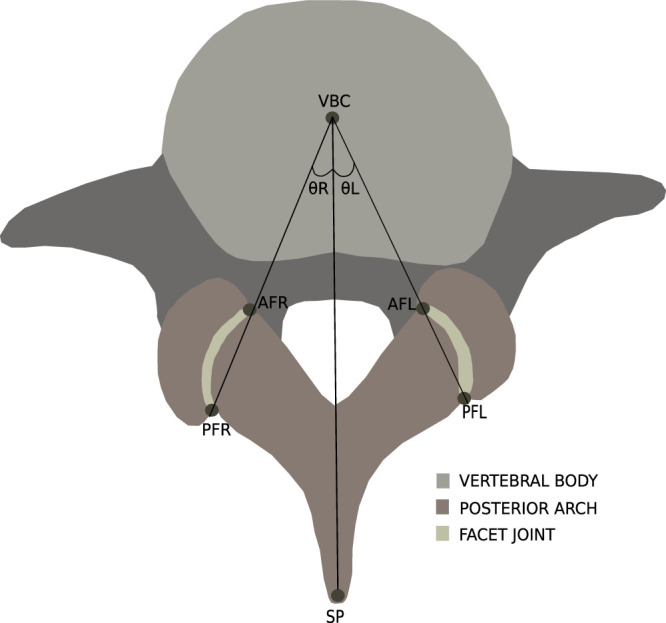
Regions of interest and facet joint angles were analyzed in the study as seen in axial spine magnetic resonance imaging slices. The points in red are the six landmarks, that is, anterior facet right (AFR), posterior facet right (PFR), anterior facet left (AFL), posterior facet right (PFR), vertebral body center (VBC), and spinous process (SP). The landmarks are used for computing the angles $\theta_{L}$ and $\theta_{R}$ .

We adopted the same method for measuring FJ angles, as described in several prior FT studies (Table 1). Specifically, the left ( $\theta_{L}$ ) and right ( $\theta_{R})$ angles were measured between the lines joining the landmark points, that is, anterior facet right, posterior facet right and anterior facet left, posterior facet left with the line joining vertebral body center, and spinous process (Fig. 2). We calculated asymmetry as the difference between the $\theta_{L}$ and $\theta_{R}$ . To clarify the definition of FT and the threshold, we conducted a thorough search in the literature accessible from Google Scholar and PubMed using the keywords “Facet Tropism,” “Facet Joint Angles,” “Zygapophyseal angles,” and included all the studies that measured angles to calculate FT. A compiled summary of studies defining different thresholds for FT is shown in Table 1.

### Facet Segmentation

To automatically segment the axial LSMRI with the identified ROI, we trained UNet++^28^—an encoder and decoder architecture widely used in medical image segmentation. Our model architecture consists of an EfficientNetB6^29^ encoder with an input image (shape 512×512, pixel spacing 0.35 mm) processed as a single-channel gray-scale image. The network output consisted of four classes: VB, FJ, PA, and the background. As the proportion of classes in terms of their size is unbalanced, we used Generalized Dice Loss^30^ as the loss function.

The data set of 430 participants was split into 370 participants for training and 60 for testing. The model was trained using the training set, which was further split into learning and validation sets at the participant level in a fivefold cross-validation setting for 200 epochs with early stopping (patience=20 epochs). Model selection and hyperparameter tuning were done based on the performance of the validation set using the intersection over union (IoU) as the metric. The model was trained with normalized input images (512×512) using a batch size of six, Adam optimizer with a weight decay of 1e-4, learning rate of 1e-4, and image augmentations (Random Tone Curve, Gamma, Brightness, Contrast, Horizontal Flip, and Gaussian Blur). Pytorch^31^ (Version: 1.12.1) framework was utilized for model development. All the experiments were conducted on 2xNVIDIA RTX A4000 Graphical Processing Units.

To evaluate the performance on the test set, we measured the overlap of the predicted segmentation masks with the ground truth annotations using the Dice score and IoU for each class individually. In addition, we assessed the distance metrics, such as average surface distance (ASD) and 95% Hausdorff distance for each class to assess the adversaries.

### Postprocessing

**Figure 3:**
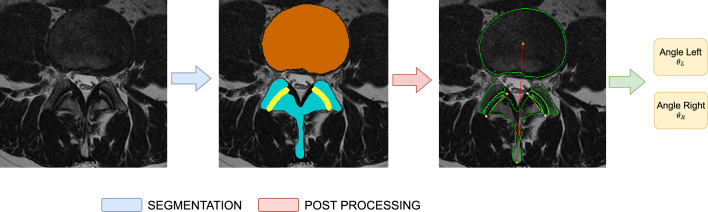
Automatic pipeline for measurement of facet joint angles. The selected axial lumbar spine T2 magnetic resonance slices are automatically processed by a deep learning model to produce segmentations. The segmentations are then used to compute the landmarks and, subsequently, to calculate the angles for left and right facet joints.

We used OpenCV^32^ (Version: 4.6.0) to compute the required landmarks (Fig. 2). The landmarks (anterior facet right, posterior facet right, anterior facet left, and posterior facet left) were computed by taking the anterior and posterior end points of the left and right FJ ROIs. The center of mass of the ROI for VB was taken as the vertebral body center, and the posterior end of the ROI for the PA was taken as the spinous process. After the landmark points were computed, the angles $\theta_{L}$ and $\theta_{R}$ were calculated for all the 1288 participants. The overall pipeline is shown in Figure 3.

### Inter-Rater and Intrarater Agreement

The rater agreement was evaluated both for the annotations and the FJ angles computed based on the annotations. FJ asymmetry ( $\theta_{L}$ - $\theta_{R})$ was used to evaluate the agreement and correlation between the raters. Bland-Altman analysis^33^ was used to assess the agreement and systemic bias in the FJ asymmetry. In addition to FJ asymmetry, we evaluated the coefficient of determination *r* ^2^ between the raters for $\theta_{L}$ and $\theta_{R}$ separately. Finally, we used the Dice score as the metric to compare the annotations between the raters. In addition, we evaluated the model predictions on the independent test set and compared them against the ground truth annotations produced by A1, A2, and A3.

## RESULTS

The model performance was evaluated using two different methods: region-based and distance metrics. While the region-based metrics (Dice, IoU) suggest that the model was able to detect the ROIs with high accuracy using just 10% of the data (Table 2), there was a noticeable improvement of 19.2% in the ASD (contour precision) and 51.9% in the 95% Hausdorff distance (distance to the adversary) for the smaller ROI, that is, FJ when 50% of the data was used (Table 3). This is essential to getting more refined contours for better computation of angles between the FJ. We observed that the model using 50% data gave a comparable performance (difference ~ 1.2%–ASD metric for FJ) to the model trained on the entire data set.

**TABLE 2:** Performance of Deep Learning-based Segmentation Model Trained on Different Data Regimens

Setting	Fivefold cross-validation		Independent test set	
	Dice	IoU	Dice	IoU
10% data	0.919±0.003	0.858±0.004	0.924±0.002	0.866±0.003
50% data	0.927±0.002	0.871±0.003	0.931±0.001	0.878±0.002
100% data	0.927±0.001	0.871±0.002	0.933±0.001	0.880±0.002

The numbers are mean and standard error of Dice score (Dice) and intersection over union (IoU) as measured on a fivefold cross-validation and an independent test set.

**TABLE 3:** Automatic Segmentation Performance Measure for Individual Regions of Interest Vertebral Body (VB), Facet Joint (FJ), and Posterior Arch (PA) at Different data Regimens

Setting	ASD VB	95HD VB	ASD FJ	95HD FJ	ASD PA	95HD PA
10% data	0.479±0.010	1.687±0.096	0.505±0.025	2.568±0.525	0.637±0.026	2.417±0.515
50% data	0.418±0.008	1.457±0.077	0.408±0.010	1.235±0.059	0.601±0.031	2.435±0.523
100% data	0.406±0.009	1.484±0.097	0.403±0.010	1.228±0.063	0.555±0.026	2.284±0.512

The numbers are the mean and SE of ASD and 95HD in millimeters.

95HD indicates 95% Hausdorff distance; ASD, average surface distance.

**Figure 4:**
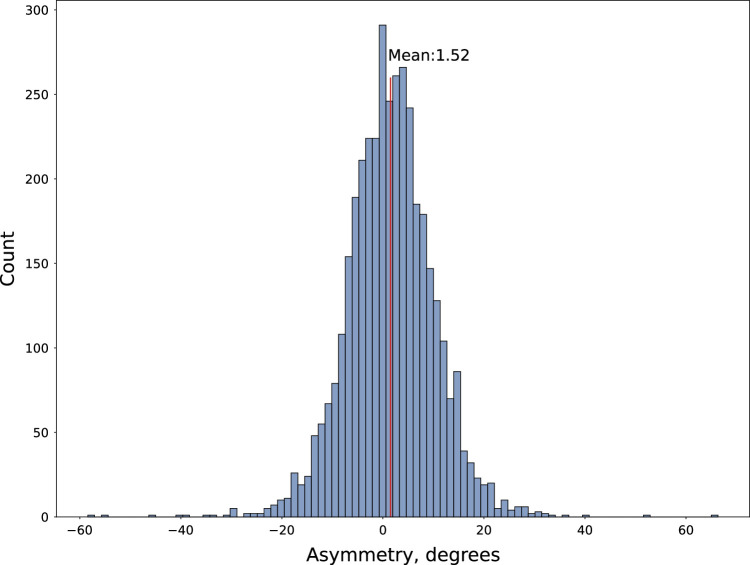
Distribution of facet joint asymmetry ( $\theta_{L}-\theta_{R}$ ) automatically measured in the NFBC1966 population. For ~80% of the population, the asymmetry is between the range of -10° and 10°.

The overall distribution of facet asymmetry in NFBC1966 data, which recorded 95.6% of births in northern Finland^26^ is visualized in Figure 4. The mean asymmetry is centered at 1.52°. Although the distribution appears to have a bell-shaped curve like the normal distribution, it failed the Kolmogorov-Smirnoff test for normality. We observed that 97.07% of the population have facet angles between the range of -20° to +20° with 80.12% of them having angles between the range of -10° and 10°, which is the threshold used for FT in several studies.^4,15,16,18,20^

We also evaluated asymmetry at different lumbar spine levels between male and female subgroups (Table 4). The average asymmetry for all the levels is found to be higher (7.1°>6.3°) in the male subgroup compared with the female subgroup. Also, the asymmetry increased by 19% from L3/4 to L4/5 (Table 4). Adopting the literature definitions FT>7° and FT>10°, we see L4/5 to be affected most (44.6% and 29.2%, respectively) in the male subgroup (Table 5).

**TABLE 4:** Automatically Measured Right and Left Facet Joints and Facet Joint Asymmetry (in Degrees)

Sex	Level	Angle right	Angle left	Average (left, right)	Kalichman *et al.* ^14^ average (left, right)	Asymmetry(left-right)
Female	L3-L4	36.15±0.35	38.03±0.34	37.09±0.34	38.11±0.99	6.09±0.18
	L4-L5	44.28±0.41	46.76±0.39	45.52±0.40	45.72±1.08	6.22±0.21
	L5-S1	48.00±0.41	48.11±0.42	48.05±0.41	48.55±1.19	6.61±0.22
Male	L3-L4	36.42±0.39	38.03±0.38	37.22±0.38	37.48±0.76	6.37±0.22
	L4-L5	44.49±0.45	47.68±0.46	46.08±0.45	46.59±0.89	7.62±0.25
	L5-S1	47.56±0.46	47.41±0.47	47.48±0.46	47.95±0.99	7.31±0.24

The numbers are the mean and SE of the biomarker at each lumbar spine level measured for 1288 participants from the NFBC1966 population-based cohort.

**TABLE 5:** Prevalence of FT in the NFBC1966 Population-Based Cohort Among Male and Female Subgroups for Different Lumbar Spine Levels L3/4 Through L5/S1

Sex	Level	FT (>7°) prevalence, n (%)	FT (>10°) prevalence, n (%)
Female	L3-L4	228 (33.6)	125 (18.4)
	L4-L5	236 (34.9)	133 (19.6)
	L5-S1	245 (36.2)	141 (20.8)
Male	L3-L4	215 (35.9)	118 (19.7)
	L4-L5	267 (44.6)	175 (29.2)
	L5-S1	243 (40.7)	158 (26.4)

The threshold for FT is taken as >7° and >10°.

FT indicates facet tropism.

**Figure 5:**
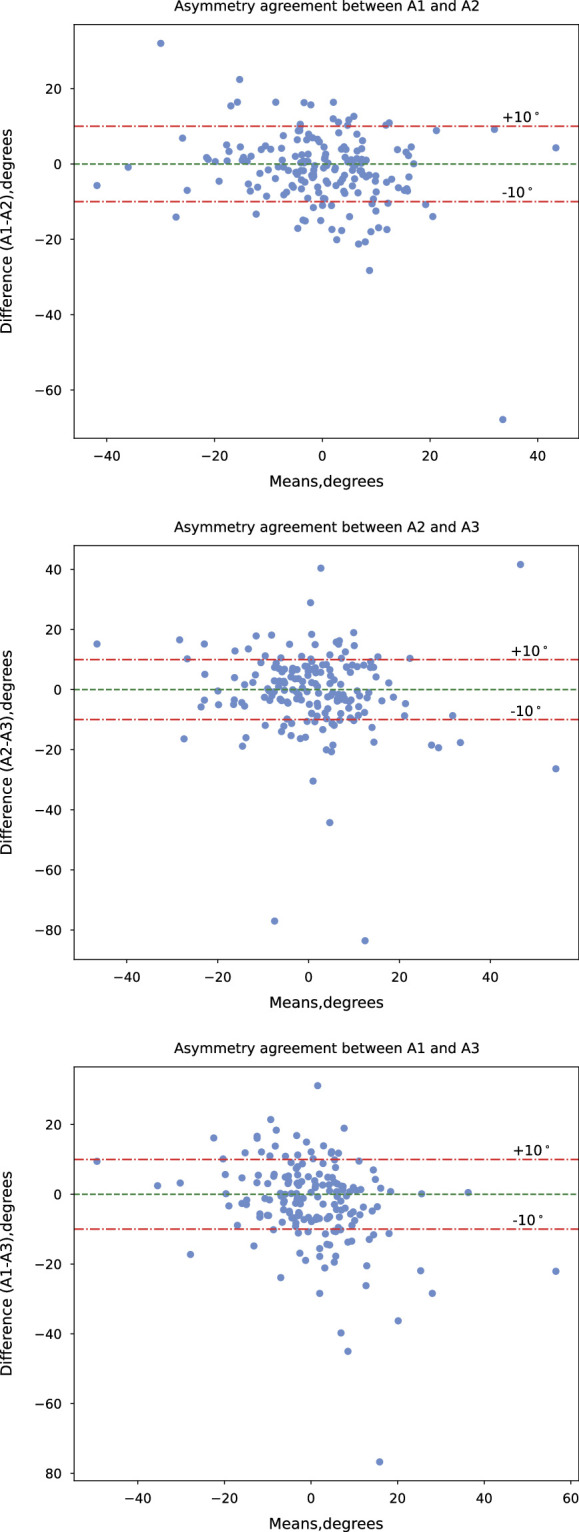
Bland-Altman agreement plots for asymmetry measurements by raters A1, A2, and A3. A higher SD is observed when comparing A1 and A2 with A3 (MSK researcher) than between A1 and A2 (radiologists), indicating the need for expert knowledge for determining facet tropism.

Neither the differences in FJ asymmetry measurements between the raters nor their log-rations followed a normal distribution based on the Shapiro-Wilk^34^ test. Therefore, we used nonparametric Bland-Altman analysis using the reference value of 10° for FT following prior research.^4,15,16,18,20^ The means and differences are then visualized between the raters A1, A2, and A3 (Fig. 5).

**Figure 6:**
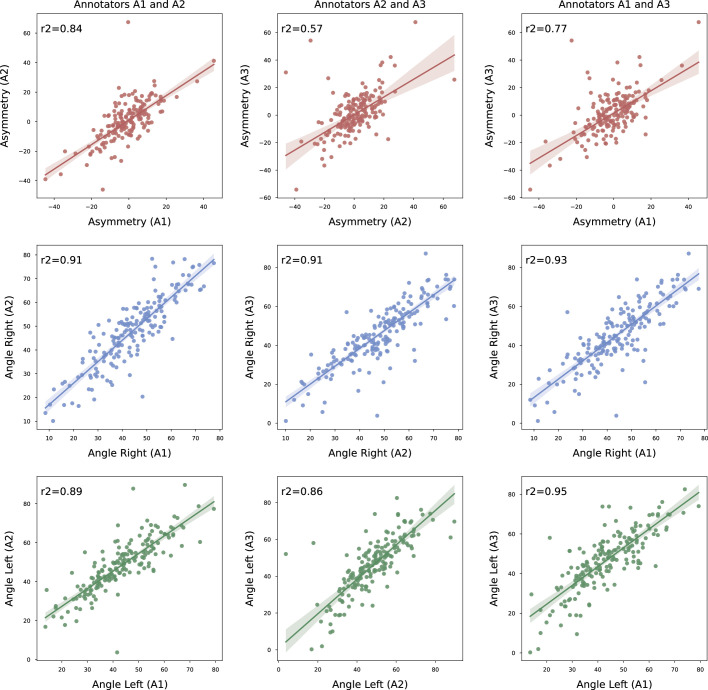
Regression plots showing the coefficient of determination *r* ^2^ for asymmetry (red), left (green), and right (blue) facet Joint angles between the raters A1, A2, and A3. The units for all the axes are in degrees. Overall correlation is higher between the radiologists than between the radiologists and MSK researcher.

A1 and A2 measurements agree more with the literature definition of FT, with 76.8% of participants falling under 10° compared with 68.3% and 66.6%, respectively, than when A1 and A2 were compared with A3. This could be partly explained by the differences in training and experience of the raters; that is, A1 and A2 are radiologists, and A3 is a MSK researcher. As seen in Figure 6, the coefficient of determination *r* ^2^ for asymmetry shows a higher (0.84) correlation between A1 and A2 (radiologists) and a lower (0.57 and 0.77, respectively) compared with A3 (MSK researcher).

**Figure 7:**
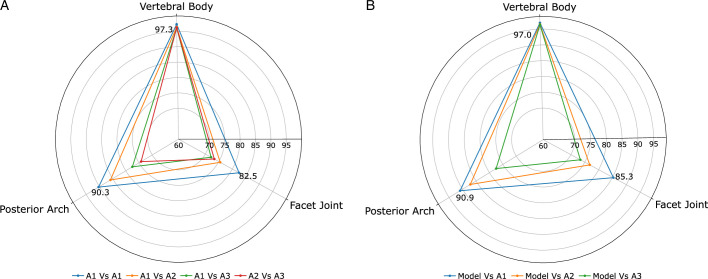
(A) Intrarater (A1-A1) and inter-rater (A1-A2, A2-A3, A1-A3) reliability assessed using Dice score per region of interest individually. (B) Similarity (Dice score) of the model predictions and the reference annotation of the raters A1, A2, and A3.

In Figure 7A, we showed the inter and intrarater reliability using Dice scores per ROI. We observed higher scores (>0.9) for PA and VB for inter-rater and intrarater agreement. The intrarater agreement for FJ is 0.82, with a maximum inter-rater agreement of 0.76 between A1 and A2. Although there is lower agreement for FJ, it does not directly translate to angle space as the correlation between A1 and A2 for asymmetry (Fig. 6) is very high (*r* ^2^=0.84). Also, in Figure 7B, we can see that the model conforms (*i.e.* learned to mimic A1 through training) to the inter-rater reliability established with the ground truth annotations (Fig. 7A), that is, if the model is labeled as A1 in Figure 7B, it closely mirrors the plot in Figure 7A.

## DISCUSSION

In this study, we presented a semiautomatic approach for measuring the FJ angles using a DL framework. We found that the average FJ angles for both male and female subgroups closely match the manual measurements performed on 188 participants using CT in a study by Kalichman *et al.* ^14^ on a different population. We hypothesized that the FJ were structurally similar across populations. Although CT provides better details for FJ,^35^ prior works used MRI to assess the degree of FT^18,36,37^ with substantial intermethod agreement when compared with CT.^35^ Therefore, this MRI-based method can be used for opportunistic screening and would not require any additional sequences, saving time and associated costs.

Currently, there is no universal threshold defined for FT (Table 1). Although we would like to define a biomarker for FT, it would only be plausible after studying its association with various LSD, considering the confounders, for example, sex, BMI, demographics, *etc*., to be clinically relevant. We postulate that the discovery of biomarkers may help us identify the risk factors for certain LSD, such as DD,^4^ degenerative lumbar spinal stenosis,^6^ *etc*. Our method can scale the research on FT as it is found to be associated with many LSD^38^ and surgical risk factors.^39^

**Figure 8:**
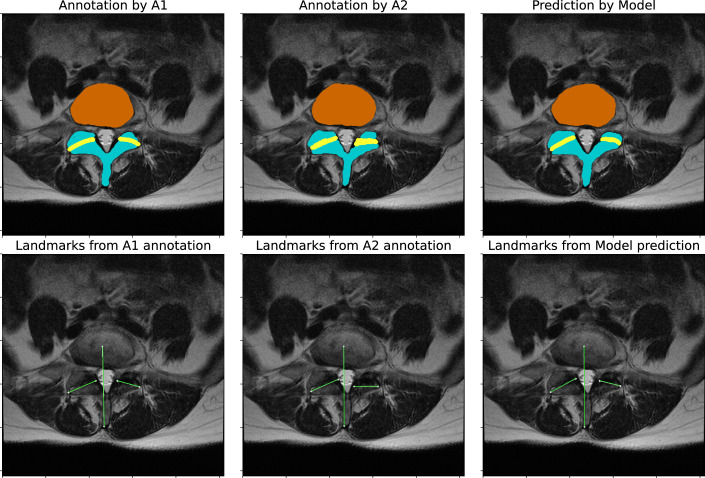
A representative sample of manual annotations shows that the two most experienced raters, A1 and A2, disagree. The landmarks for the facet joint on the left (patient-left), as annotated by A2, are more inclined toward the sagittal plane, resulting in a large difference in angle estimates. The model predictions conform to A1 since it is trained on A1 annotations.

Another observation was that the measurement of facet angles can be sensitive to the ground truth labels used for training. For example, in Figure 8, we can see that the ground truth label for the left FJ was annotated differently by A1 and A2. As a result, there is a notable difference in the $\theta_{L}$ as compared with the $\theta_{R}$ . We believe that this problem is not directly associated with the method but can be seen as a subjective difference in the landmark points of the FJ, which would occur even if the angles were manually measured.^35^ One possible solution is to reach a consensus agreement between the raters. However, this would translate to more annotations required per rater, with the challenge of reproducibility.^40^

There are several limitations to this study. First, the DL model is trained on images from a single center and annotations from a single rater (Fig. 1). This is due to the general availability and disproportionate data. Although we observed a high inter-rater correlation in asymmetry (*r* ^2^=0.84) between radiologists (Fig. 6), future works may benefit from incorporating methods that account for raters’^41,42^ and center^43^ variability. Second, the demographics of NFBC1966 are confined to the Finnish population and may not account for the variation of asymmetry in relation to the subject’s age, race, *etc*. Third, our study lacks participant data identifying FJ disorders (*e.g.* FJ osteoarthritis and osteophytes) that may act as confounders. Although the model is expected to generalize from the learned set of annotations for the FJ, it is important to account for these confounders in future work. Fourth, the scope of the study was to establish a method to measure FJ angles at scale using DL. So, the axial slices are currently identified manually per level, making it a semiautomatic framework. Therefore, the model performance is evaluated in a less-than-perfect condition and can contribute to some errors in the measurement of FT. In future research, we would fully automate the method and include diverse populations to create a plausible biomarker for FT.

In conclusion, here we present the first DL-based method for large-scale analyses of FT in a population-based cohort using LSMRI data. Using the proposed method, we present the prevalence of FT in a Finnish population-based cohort. We hope that downstream applications of our method will shed more light on FT and its clinical relationship with LSD.

Key Points

- We developed a deep learning-based method to measure the facet joint (FJ) angles.
- The reliability of the developed model conforms to the reliability of the human raters.
- Inter-rater reliability showed considerable variability between raters with diverse backgrounds.
- The FJ angles measured in the Finnish population-based cohort closely match prior literature, indicating a possible similarity in the FJ orientation across the populations.
